# Validation of the Chinese version of the Rosenberg Self-Esteem Scale: evidence from a three-wave longitudinal study

**DOI:** 10.1186/s40359-023-01293-1

**Published:** 2023-10-18

**Authors:** Chen Jiang, Yihong Zhu, Yi Luo, Chee-Seng Tan, Stefanos Mastrotheodoros, Patrício Costa, Li Chen, Lina Guo, Haiyan Ma, Runtang Meng

**Affiliations:** 1 School of Public Health, Hangzhou Normal University, Hangzhou, 311121 Zhejiang China; 2 School of Clinical Medicine, Hangzhou Normal University, Hangzhou, Zhejiang China; 3 School of Nursing, Ningbo College of Health Sciences, Ningbo, Zhejiang China; 4 School of Psychology, College of Liberal Arts, Wenzhou-Kean University, Wenzhou, Zhejiang China; 5 Department of Psychology and Counselling, Faculty of Arts and Social Science, Universiti Tunku Abdul Rahman, Kampar, Perak Malaysia; 6 Department of Psychology, University of Crete, Rethymno, Greece; 7 Department of Youth and Family, Utrecht University, Utrecht, the Netherlands; 8 Life and Health Sciences Research Institute (ICVS), School of Medicine, University of Minho, Braga, Portugal; 9 ICVS/3B’s – PT Government Associate Laboratory, Braga/Guimarães, Portugal; 10 Faculty of Psychology and Education Sciences, University of Porto, Porto, Portugal; 11 Digestive System Department, Yan’an Hospital of Kunming City, Kunming, Yunnan China; 12 Department of Neurology, The First Affiliated Hospital of Zhengzhou University, Zhengzhou, Henan China; 13 Engineering Research Center of Mobile Health Management System, Ministry of Education, Hangzhou, Zhejiang China

**Keywords:** Rosenberg Self-Esteem Scale, Validation, Psychometric properties, Measurement invariance, Longitudinal study

## Abstract

**Background:**

The 10-item Rosenberg Self-Esteem Scale (RSES) is a widely used tool for individuals to self-report their self-esteem; however, the factorial structures of translated versions of the RSES vary across different languages. This study aimed to validate the Chinese version of the RSES in the Chinese mainland using a longitudinal design.

**Methods:**

A group of healthcare university students completed the RSES across three waves: baseline, 1-week follow-up, and 15-week follow-up. A total of 481 valid responses were collected through the three-wave data collection process. Exploratory factor analysis (EFA) was performed on the baseline data to explore the potential factorial structure, while confirmatory factor analysis (CFA) was performed on the follow-up data to determine the best-fit model. Additionally, the cross-sectional and longitudinal measurement invariances were tested to assess the measurement properties of the RSES for different groups, such as gender and age, as well as across different time points. Convergent validity was assessed against the Self-Rated Health Questionnaire (SRHQ) using Spearman’s correlation. Internal consistency was examined using Cronbach’s alpha and McDonald’s omega coefficients, while test–retest reliability was assessed using intraclass correlation coefficient.

**Results:**

The results of EFA revealed that Items 5, 8, and 9 had inadequate or cross-factor loadings, leading to their removal from further analysis. Analysis of the remaining seven items using EFA suggested a two-factor solution. A comparison of several potential models for the 10-item and 7-item RSES using CFA showed a preference for the 7-item form (RSES-7) with two factors. Furthermore, the RSES-7 exhibited strict invariance across different groups and time points, indicating its stability and consistency. The RSES-7 also demonstrated adequate convergent validity, internal consistency, and test–retest reliability, which further supported its robustness as a measure of self-esteem.

**Conclusions:**

The findings suggest that the RSES-7 is a psychometrically sound and brief self-report scale for measuring self-esteem in the Chinese context. More studies are warranted to further verify its usability.

**Supplementary Information:**

The online version contains supplementary material available at 10.1186/s40359-023-01293-1.

## Background

Self-esteem is considered to be a set of thoughts and feelings about one’s self-worth and importance; that is, a global positive or negative attitude towards the self [1]. Positive self-esteem is often regarded as a protective factor for mental health and a buffer against adverse events [2, 3]. Conversely, negative self-esteem is seen as a risk factor for psychiatric disorders and social problems [4–8]. Arguably, self-esteem is a highly crucial psychological need that requires the attention and protection of each individual as well as wider society; therefore, it is essential to gain a deeper understanding of its subjective evaluation.

To date, the 10-item Rosenberg Self-Esteem Scale (RSES), developed in 1965 [1], is one of the most accepted and globally used scales for measuring self-esteem. It has been translated into more than 28 languages and used in 53 countries and regions, and this data continues to grow [9]. Rosenberg proposed that people with high self-esteem tend to be self-respecting, consider themselves worthy, and appreciate their own merits while recognizing their faults. People with low self-esteem lack respect for themselves and consider themselves to be unworthy, inadequate, or seriously deficient [10, 11]. Regarding its measurement, unlike many other scales that assess self-esteem, the RSES is concise and convenient [9, 12]. The low number of items, short completion time, and reduced chance of respondent tiredness facilitate its ease of use in various cohorts.

The RSES has been translated into numerous languages since it was first developed [13–15]. Even though many studies have supported the psychometric properties of the different versions, such as the Spanish, German, Dutch, and Japanese versions [16–18], there is ongoing controversy about whether the RSES is unidimensional or multidimensional and whether the difference between positive and negative self-esteem is due to language effects [19]. In cross-cultural validation, many studies have reported low factor loadings for some items, an unstable factor structure, and a cross-cultural misfit [20–23]. More importantly, cultural differences between the East and West, caused by different understandings of negatively worded items, may have confined the cross-cultural comparisons [9].

Several studies have examined the psychometric properties of different Chinese versions of the RSES. In 1993, the first translation in simplified Chinese resulted in a version of the RSES that showed poor reliability [24]. In that study, Item 8 (“I wish I could have more respect for myself”) resulted in a negative item-total correlation due to translation bias and cultural differences [24]. Other researchers have discussed the removal of Item 8 yet failed to reach a consensus [25–27]. In 1997, a version in traditional Chinese was created in Hong Kong, China, to provide a self-esteem instrument for Cantonese-speaking people [28]. Given the unsatisfactory reliability (*N* = 1101, Cronbach’s alpha = 0.686) of this version, scholars in Macau, China, modified Items 2, 3, 7, and 8 to adapt the RSES to the local culture [29]. The adaptations resulted in a version with improved scale reliability, although Item 8 retained suboptimal psychometric properties [29]. After comparison, we chose the traditional Chinese adaptation for use in the current study, which was conducted in the Chinese mainland after the traditional Chinese adaptation was converted directly into simplified Chinese.

Since societal processes influence self-esteem, it is crucial to assess whether different versions of the RSES work in a similar way across different contexts and generations. Thus, a longitudinal study focusing on the utility of the simplified Chinese adaptation of the RSES within the Chinese mainland context can provide new evidence to the extant literature and ongoing exploration of the Chinese version. The goal of this study, which was with a Chinese healthcare students cohort, was mainly twofold: (i) evaluate the main psychometric properties of the scale—structural validity, convergent validity, internal consistency, and test–retest reliability; (ii) test the cross-sectional and longitudinal measurement invariance.

## Methods

### Study design and procedure

The study used a three-wave longitudinal observational design among healthcare students in Hangzhou, China. The protocol adhered strictly to the STrengthening the Reporting of OBservational studies in Epidemiology (STROBE) guidelines to ensure the accurate, high-quality presentation of the research [30].

Minimum sample size guidelines recommend 15 participants per variable; hence, as there are 10 items in the RSES, the required sample was 150 [31]. Using a stratified random sampling method, healthcare students in the medical department of one university in Hangzhou were randomly selected to participate in a paper-and-pencil survey from December 2020 to April 2021. Before the survey, we contacted the leaders of the target classes to determine when the respondents would have free time and subsequently conducted the survey in the classroom during breaks. We collected student ID numbers; this step was for matching the same individual across three waves. A total of 637 healthcare students participated in the initial baseline assessment. One week later, 616 students underwent the re-assessment wave [32, 33]. After a 15-week interval, 540 students completed the third assessment. There data from 512 participants were successfully matched across three waves; after participants with missing data were removed from the dataset, 481 individuals were left for the subsequent analysis. This study was approved by the Institutional Review Board of Hangzhou Normal University Division of Health Sciences, China (Reference No. 20190076). The data collection process with prior informed consent was undertaken anonymously to protect individual privacy rights.

### Measures

#### Rosenberg Self-Esteem Scale

The RSES [1] consists of five positively worded items (1, 3, 4, 7, 10) and five negatively worded items (2, 5, 6, 8, 9), and serves as one of the most broadly used instruments for global self-esteem. The scale was initially designed to be unidimensional, yet numerous studies worldwide have revealed that it may be multidimensional, with both positive and negative self-esteem dimensions. Positively worded items are given a score from 1 (strongly agree) to 4 (strongly disagree). Negatively worded are reverse scored, from 1 (strongly disagree) to 4 (strongly agree). The total sum score for all 10 items ranges from 10 to 40, with higher scores representing higher self-esteem. The scale used in this study was the traditional Chinese language adaptation, developed in Macau, China [29], that was converted into simplified Chinese for the purposes of this study.

#### Self-Rated Health Questionnaire

The Self-Rated Health Questionnaire (SRHQ) [34] is a two-item scale that assesses physical and psychological health. Participants reported their health status on a five-point Likert scale with varying response categories (1 = excellent, 2 = good, 3 = average, 4 = poor, 5 = extremely poor), giving a total sum score ranging from 2 to 10. Higher scores represent poorer overall self-rated health. The scale has shown stable psychometric properties in recent measurements with large samples (Cronbach’s alpha = 0.706) [34].

#### Sociodemographic description

The following variables were also collected: gender (0–male, 1–female), age (mean = 19.688, standard deviation = 1.329), home location (0–urban, 1–rural, 2–suburban), single-child status (0–yes, 1–no), academic year (0–first year, 1–second year, 2–third year), family income (0– < 10 000 CNY, 1– ≥ 10 000 CNY), part-time employment (0–yes, 1–no), leisure-time sports involvement (0–yes, 1–no).

### Statistical analysis

Measurement properties were assessed based on the COnsensus-based Standards for selecting health Measurement INstruments guidelines (COSMIN) [35, 36]. EpiData (version 3.1), JASP (version 0.16.1), and R (version 4.1.2) software were used for database creation, data organization, and data analysis, respectively. Missing data analysis was performed using the “*naniar*” package and showed that out of the 512 participants who completed the questionnaires on all three occasions, 481 (93.945%) had no missing values, and 31 (6.055%) had missing values. The missing data rate for the RSES items and sample variables ranged from 0.195% to 1.758%. Listwise deletion was applied since the level of missing data was negligible in this study [37]. The multivariate normality test of scores was performed using the “*MVN* v.5.9” package [38].

### Structural validity

To assess the structural validity of the RSES, exploratory factor analysis (EFA) was performed on the baseline, and confirmatory factor analysis (CFA) was performed on the 1-week and 15-week follow-ups using the “*lavaan* v.0.6–9” package [39]. Before EFA, item-total correlation, two tests, Kaiser–Meyer–Olkin (KMO, KMO ≥ 0.800) and Bartlett’s test (*P* < 0.001), were implemented to examine the factorability of the data [40, 41]. EFA with the weighted least squares mean and variance adjusted (WLSMV) method, Promax rotation, and parallel analysis was used for the factor extraction. When the target-loading was less than 0.450, the cross-loading was higher than 0.320, or the gap between the target-loading and cross-loading was lower than or equal to 0.200, the item was considered for removal [41, 42].

Given the ordinal nature of the variables, in the CFA we chose the WLSMV estimator, which shows less bias in standard errors and yields more accurate factor loadings [43]. Fit indices were considered to be acceptable when they were within the following thresholds: Chi-square/degree of freedom (*χ*^*2*^*/df*) = 2‒3, comparative fit index (CFI) ≥ 0.900, Tucker-Lewis index (TLI) ≥ 0.900, standardized root mean residual (SRMR) ≤ 0.080, root mean square error of approximation (RMSEA) ≤ 0.080 [37, 44, 45].

### Measurement invariance

The measurement invariance of the RSES was examined by comparing five nested models (i.e., configural, threshold, metric, scalar, and strict invariance model) with progressively tighter restrictions using the “*semTools* v.0.5–5” package [46]. A range of tests were conducted: configural invariance tests assessed whether the constellation of items and factors was the same across groups or time; threshold invariance tests assessed whether the association of the underlying (latent) continuous score with the ordinal numbers of the items was the same across groups or time; metric invariance tests whether the factor loadings of each item were the same across groups or time; scalar invariance tests assessed whether the item intercepts were the same across groups or time; and finally, strict invariance was used to examine whether the error variance (residuals) of each item were the same across groups or time.

To comprehensively examine the scale’s usability, we analyzed the cross-sectional measurement invariances (CMIs) in the best-fit scale model across gender and age. This was because previous research has shown different in self-esteem between genders and age groups [47]. We also examined the measurement invariance across home location, single-child status, academic year, family income, part-time employment, and leisure-time sports involvement to explore their potential influence (if any) on self-esteem measurement.

To test for response shifts, through longitudinal CFA, the longitudinal measurement invariances (LMIs) were analyzed across three waves: baseline, 1-week follow-up, and 15-week follow-up. Measurement invariance was assumed when two of the three following indices met the criteria: ΔCFI ≤ 0.010, ΔTLI ≤ 0.010, ΔRMSEA ≤ 0.015 [48–50].

### Convergent validity

Spearman’s correlation was used to examine convergent validity by testing the correlation between two relevant constructs. Given that self-esteem measured by the RSES has been associated with self-rated mental health using the SRHQ, a moderately strong correlation (-0.500 ≤ *r* ≤ -0.300) between the SRHQ and RSES was hypothesized. Meanwhile, the average variance extracted (AVE; AVE > 0.500) and construct reliability (CR; CR > 0.700) were also integrated to assess convergent validity [51].

### Internal consistency

The internal consistency of the subscale and total scores for the RSES and SRHQ across the three waves was assessed by calculating Cronbach’s alpha (α) and MacDonald’s omega (ω) using the “*ufs* v.0.4.5” package in R [52, 53]. Cronbach’s α is the most commonly used coefficient; however, in consideration of its reported imperfections, MacDonald’s ω was calculated simultaneously to provide more objective confidence estimates [53]. Both α and ω were considered acceptable when ≥ 0.700 [36, 53–55].

### Test–retest reliability

Test–retest reliability was assessed using the intraclass correlation coefficient (ICC), with ICC ≥ 0.700 considered as the preferable value [56]. Standard error of measurement was also computed using “standard deviation × sqrt (1-ICC)”. The test–retest reliability was performed using the *“irr* v.0.84.1” package in R [57].

## Results

### Sample characteristics

The final sample size for this study was 481. The participant characteristics and the RSES total scores for the three measurement waves are presented in Supplementary Material, Table S1.

### Structural validity

The results of the KMO test (KMO = 0.900) and Bartlett’s test (*χ*^*2*^ = 1976.017, *df* = 45, *P* < 0.001) for the 10-item RSES (RSES-10) suggested that the scale was suitable for factor analysis. EFA of the baseline data revealed two factors (see Table 1). However, the factor loading for Item 8 (“I wish I could have more respect for myself”) was below 0.450; hence, it was removed. Subsequent EFA of the remaining nine items suggested removing Item 5 (“I feel I do not have much to be proud of”) due to a factor loading below 0.450, and then removing Item 9 (“All in all, I am inclined to feel that I am a failure”) due to a gap between the target-loadings and cross-loadings of below 0.200. The results of the 7-item RSES (RSES-7) without Items 5, 8, and 9 (KMO = 0.848; *χ*^*2*^ = 1336.556, *df* = 21, *P* < 0.010) revealed two factors and accounted for 57.6% of the total variance. The factor loadings for the positive (0.577 to 0.812) and negative (0.597 to 1.052) subscales were acceptable.

**Table 1:** EFA factor loadings: RSES-10 and RSES-7

Variable	Positive	Negative	KMO test	Bartlett’s test	Cumulative variance
RSES-10			0.900	1976.017 (45) ***	0.500
RSES01	0.703	-0.033			
RSES02	-0.055	0.882			
RSES03	0.712	0.021			
RSES04	0.807	-0.131			
**RSES05**	**0.442**	**0.261**			
RSES06	0.015	0.801			
RSES07	0.794	-0.088			
*RSES08*	*-0.051*	*0.381*			
**RSES09**	**0.481**	**0.324**			
RSES10	0.577	0.141			
RSES-7			0.848	1336.556 (21) **	0.576
RSES01	0.705	-0.021			
RSES02	0.179	0.597			
RSES03	0.710	0.022			
RSES04	0.812	-0.120			
RSES06	-0.093	1.052			
RSES07	0.759	-0.040			
RSES10	0.577	0.110			

Bold font indicates items with cross-loading. Italics indicates items with low factor loading

Abbreviations: *EFA* Exploratory factor analysis, *RSES* Rosenberg Self-Esteem Scale, *KMO test* Kaiser-Meyer-Olkin test

***P < 0.001

**P < 0.010

As the factor loading of Item 6 exceeded one, we also explored another model without Item 6. Again, a two-factor solution was found. However, the negative factor only comprised one item (Item 2). After removing this single item and rerunning the EFA, the five positively worded items loaded onto a single factor and explained 50% of the total variance (see Supplementary Material, Table S2).

Several CFAs were then conducted to examine the following models for the RSES-10 and RSES-7: a one-factor model, a two-factor model (with positive and negative factors), a second-order factor model (with a general factor of self-esteem accounting for the two specific factors), and a two-factor model for acquiescence (with a general factor of self-esteem and a method factor of acquiescence). The same analyses were conducted with the data collected from the 1-week follow-up and 15-week follow-up. As can be seen in Table 2, the two-factor model was superior to the other three models for both the RSES-10 and RSES-7. The same pattern of results was also observed in both follow-up datasets. Finally, inspection of the two-factor RSES-10 and RSES-7 models demonstrated found that the RSES-7 showed a better fit, and the two-factor model for acquiescence indicated that the difference between the two models was not caused by the method. In other words, the results suggest that the 7-item simplified Chinese language RSES with two factors was the preferable model.

**Table 2:** CFA outcomes: RSES-10 and RSES-7

Form	Model	*χ*^*2*^	*df*	CFI	TLI	SRMR	RMSEA (90% CI)
RSES-10	1-week follow-up						
	One-factor Model	486.462	35	0.963	0.952	0.084	0.164 (0.151, 0.177)
	**Two-factor Model**	**337.675**	**34**	**0.975**	**0.967**	**0.061**	**0.136 (0.123, 0.150)**
	Second-order factor Model	1498.160	35	0.879	0.844	0.151	0.295 (0.282, 0.308)
	Two-factor Model for acquiescence	798.407	39	0.208	0.086	0.295	0.201 (0.189, 0.214)
	15-week follow-up						
	One-factor Model	456.995	35	0.956	0.944	0.071	0.158 (0.146, 0.172)
	**Two-factor Model**	**270.817**	**34**	**0.976**	**0.968**	**0.051**	**0.120 (0.107, 0.134)**
	Second-order factor Model	1434.081	35	0.856	0.814	0.139	0.289 (0.276, 0.301)
	Two-factor Model for acquiescence	789.003	39	0.233	0.115	0.277	0.200 (0.188, 0.212)
RSES-7	1-week follow-up						
	One-factor Model	358.825	14	0.959	0.939	0.105	0.227 (0.207, 0.247)
	**Two-factor Model**	**83.039**	**13**	**0.992**	**0.987**	**0.042**	**0.106 (0.085, 0.128)**
	Second-order factor Model	256.766	14	0.971	0.957	0.104	0.190 (0.170, 0.211)
	Two-factor Model for acquiescence	436.146	15	0.371	0.119	0.243	0.235 (0.216, 0.254)
	15-week follow-up						
	One-factor Model	316.597	14	0.956	0.935	0.076	0.212 (0.192, 0.233)
	**Two-factor Model**	**31.680**	**13**	**0.997**	**0.996**	**0.022**	**0.055 (0.031, 0.079)**
	Second-order factor Model	264.712	14	0.964	0.946	0.092	0.193 (0.173, 0.214)
	Two-factor Model for acquiescence	437.655	15	0.360	0.104	0.232	0.235 (0.216, 0.254)
	Threshold			≥ 0.900	≥ 0.900	≤ 0.080	≤ 0.080

Bold font stands for the best fit model

Abbreviations: *RSES* Rosenberg Self-Esteem Scale, *χ*^*2*^ Chi-square, *df* degrees of freedom, *CFI* comparative fit index, *TLI* Tucker-Lewis index, *SRMR* standardized root mean residual, *RMSEA* root mean square error of approximation, *CI* confidence interval

### Measurement invariance

#### Cross-sectional measurement invariance

Table 3 summarizes the CMI results for the RSES-7 across eight subgroups (e.g., gender, age, family income) for the three waves. The results showed that at least two of the three indices (ΔCFI, ΔTLI, and ΔRMSEA) in each subgroup met the suggested criteria, indicating that there were negligible changes between two adjacent models [58]. Thus, the threshold, metric, scalar, and strict invariance models were all supported for the RSES-7.

**Table 3:** Cross-sectional measurement invariances: RSES-7 with two factors

Hypothesis	*χ*^*2*^ (*df*)	Δ*χ*^*2*^ (Δ*df*)	CFI	ΔCFI	TLI	ΔTLI	RMSEA	ΔRMSEA	
**Gender (Male vs. Female)**									
* Baseline*									
Configural Model	71.127 (26) ***		0.990		0.984		0.085 (0.062, 0.109)		
Threshold Model	71.516 (31) ***	4.074 (5)	0.991	0.001	0.988	0.004	0.074 (0.051, 0.096)	-0.011	
Metric Model	84.741 (36) ***	9.806 (5)	0.989	-0.002	0.987	0.000	0.075 (0.055, 0.096)	0.001	
Scalar Model	92.103 (41) ***	6.442 (5)	0.989	-0.001	0.988	0.001	0.072 (0.052, 0.092)	-0.003	
Strict Model	113.673 (48) ***	15.000 (7) *	0.985	-0.003	0.987	-0.001	0.076 (0.058, 0.094)	0.003	
* 1-week follow-up*									
Configural Model	90.344 (26) ***		0.993		0.988		0.102 (0.079, 0.125)		
Threshold Model	88.703 (32) ***	6.225 (6)	0.994	0.001	0.992	0.003	0.086 (0.065, 0.108)	-0.016	
Metric Model	103.678 (37) ***	12.398 (5) *	0.993	-0.001	0.992	0.000	0.087 (0.067, 0.107)	0.001	
Scalar Model	109.693 (42) ***	4.243 (5)	0.992	0.000	0.992	0.001	0.082 (0.063, 0.101)	-0.005	
Strict Model	107.236 (49) ***	5.973 (7)	0.994	0.001	0.994	0.002	0.070 (0.052, 0.089)	-0.012	
* 15-week follow-up*									
Configural Model	48.261 (26) **		0.996		0.994		0.060 (0.032, 0.086)		
Threshold Model	47.353 (28) *	1.009 (2)	0.997	0.000	0.995	0.001	0.054 (0.025, 0.080)	-0.006	
Metric Model	56.326 (33) **	7.351 (5)	0.996	-0.001	0.995	0.000	0.054 (0.028, 0.078)	0.001	
Scalar Model	60.124 (38) *	4.211 (5)	0.996	0.000	0.996	0.001	0.049 (0.023, 0.072)	-0.005	
Strict Model	93.124 (45) ***	21.966 (7) **	0.992	-0.004	0.992	-0.004	0.067 (0.047, 0.086)	0.018	
**Age (< 20 vs. ≥ 20)**									
*Baseline*									
Configural Model	75.539 (26) ***		0.988		0.981		0.090 (0.067, 0.114)		
Threshold Model	81.401 (31) ***	6.977 (5)	0.988	0.000	0.984	0.003	0.083 (0.061, 0.105)	-0.007	
Metric Model	83.735 (36) ***	4.514 (5)	0.989	0.001	0.987	0.003	0.075 (0.054, 0.096)	-0.008	
Scalar Model	89.168 (41) ***	4.634 (5)	0.989	0.000	0.988	0.001	0.070 (0.050, 0.090)	-0.004	
Strict Model	95.898 (48) ***	8.587 (7)	0.989	0.000	0.990	0.002	0.065 (0.046, 0.084)	-0.006	
* 1-week follow-up*									
Configural Model	100.944 (26) ***		0.991		0.986		0.110 (0.088, 0.133)		
Threshold Model	94.065 (32) ***	4.221 (6)	0.993	0.002	0.990	0.005	0.090 (0.070, 0.112)	-0.020	
Metric Model	97.039 (37) ***	3.018 (5)	0.993	0.000	0.992	0.002	0.083 (0.063, 0.103)	-0.008	
Scalar Model	102.502 (42) ***	2.997 (5)	0.993	0.000	0.993	0.001	0.078 (0.059, 0.097)	-0.005	
Strict Model	124.375 (49) ***	20.443 (7) **	0.991	-0.002	0.992	0.000	0.081 (0.063, 0.098)	0.003	
* 15-week follow-up*									
Configural Model	46.349 (26) **		0.997		0.995		0.057 (0.029, 0.084)		
Threshold Model	46.178 (28) *	0.488 (2)	0.997	0.000	0.996	0.001	0.052 (0.022, 0.078)	-0.005	
Metric Model	56.616 (33) **	9.311 (5)	0.996	-0.001	0.995	0.000	0.055 (0.029, 0.079)	0.003	
Scalar Model	58.491 (38) *	1.151 (5)	0.997	0.001	0.996	0.001	0.048 (0.020, 0.071)	-0.007	
Strict Model	72.401 (45) **	12.56 (7)	0.996	-0.001	0.996	0.000	0.051 (0.027, 0.072)	0.003	
**Home location (Urban vs. Rural vs. Suburban)**									
*Baseline*									
Configural Model	58.352 (26) ***		0.993		0.988		0.072 (0.047, 0.097)		
Threshold Model	59.578 (31) **	4.468 (5)	0.993	0.001	0.991	0.003	0.062 (0.038, 0.086)	-0.010	
Metric Model	68.623 (36) **	4.468 (5)	0.993	-0.001	0.991	0.000	0.062 (0.039, 0.083)	-0.001	
Scalar Model	81.684 (41) ***	12.062 (5) *	0.991	-0.002	0.990	-0.001	0.064 (0.044, 0.085)	0.003	
Strict Model	124.061 (48) ***	31.051 (7) ***	0.983	-0.008	0.985	-0.006	0.081 (0.064, 0.099)	0.017	
* 1-week follow-up*									
Configural Model	93.639 (26) ***		0.992		0.986		0.104 (0.082, 0.127)		
Threshold Model	89.672 (30) ***	2.426 (4)	0.993	0.001	0.990	0.003	0.091 (0.070, 0.113)	-0.013	
Metric Model	92.048 (35) ***	3.449 (5)	0.993	0.000	0.991	0.002	0.082 (0.062, 0.103)	-0.009	
Scalar Model	95.814 (40) ***	2.075 (5)	0.993	0.000	0.993	0.001	0.076 (0.057, 0.096)	-0.006	
Strict Model	109.815 (47) ***	14.115 (7) *	0.992	-0.001	0.993	0.000	0.075 (0.057, 0.093)	-0.002	
* 15-week follow-up*									
Configural Model	32.628 (26) *		0.999		0.998		0.033 (0.000, 0.064)		
Threshold Model	35.270 (28) *	2.446 (2)	0.999	0.000	0.998	0.000	0.033 (0.000, 0.063)	0.000	
Metric Model	36.647 (33) *	2.446 (2)	0.999	0.001	0.999	0.001	0.021 (0.000, 0.054)	-0.011	
Scalar Model	44.677 (38) *	7.982 (5)	0.999	-0.001	0.999	0.000	0.027 (0.000, 0.055)	0.006	
Strict Model	66.584 (45) **	15.934 (7) *	0.996	-0.002	0.997	-0.002	0.045 (0.018, 0.066)	0.018	
**Single-child status (Yes vs. No)**									
*Baseline*									
Configural Model	86.334 (26) ***		0.986		0.978		0.098 (0.076, 0.122)		
Threshold Model	88.561 (31) ***	6.175 (5)	0.987	0.001	0.982	0.004	0.088 (0.067, 0.110)	-0.010	
Metric Model	89.733 (36) ***	2.445 (5)	0.988	0.001	0.986	0.004	0.079 (0.059, 0.100)	-0.009	
Scalar Model	97.109 (41) ***	6.460 (5)	0.987	-0.001	0.987	0.001	0.076 (0.056, 0.095)	-0.003	
Strict Model	117.808 (48) ***	18.594 (7) *	0.984	-0.003	0.986	-0.001	0.078 (0.060, 0.096)	0.002	
* 1-week follow-up*									
Configural Model	110.989 (26) ***		0.990		0.983		0.117 (0.095, 0.140)		
Threshold Model	110.003 (30) ***	3.915 (4)	0.990	0.001	0.986	0.003	0.106 (0.085, 0.127)	-0.011	
Metric Model	113.951 (35) ***	4.336 (5)	0.990	0.000	0.989	0.002	0.097 (0.078, 0.117)	-0.008	
Scalar Model	117.575 (40) ***	1.121 (5)	0.991	0.000	0.990	0.002	0.090 (0.071, 0.109)	-0.007	
Strict Model	139.355 (47) ***	19.346 (7) **	0.989	-0.002	0.990	0.000	0.091 (0.073, 0.108)	0.001	
* 15-week follow-up*									
Configural Model	32.628 (26) *		0.999		0.998		0.033 (0.000, 0.064)		
Threshold Model	35.270 (28) *	2.446 (2)	0.999	0.000	0.998	0.000	0.033 (0.000, 0.063)	0.000	
Metric Model	36.647 (33) *	1.975 (5)	0.999	0.001	0.999	0.001	0.021 (0.000, 0.054)	-0.011	
Scalar Model	44.677 (38) *	7.982 (5)	0.999	-0.001	0.999	0.000	0.027 (0.000, 0.055)	0.006	
Strict Model	66.584 (45) **	15.934 (7) *	0.996	-0.002	0.997	-0.002	0.045 (0.018, 0.066)	0.018	
**Academic year (First year vs. Second year vs. Third year)**									
*Baseline*									
Configural Model	83.809 (39) ***		0.989		0.983		0.085 (0.060, 0.110)		
Threshold Model	92.100 (47) ***	9.443 (8)	0.989	0.000	0.986	0.003	0.078 (0.054, 0.101)	-0.007	
Metric Model	97.560 (57) **	8.563 (10)	0.990	0.001	0.989	0.004	0.067 (0.043, 0.089)	-0.011	
Scalar Model	110.206 (67) **	11.645 (10)	0.990	-0.001	0.990	0.001	0.064 (0.041, 0.084)	-0.003	
Strict Model	151.372 (81) ***	30.339 (14) **	0.983	-0.006	0.987	-0.003	0.074 (0.055, 0.092)	0.010	
* 1-week follow-up*									
Configural Model	135.340 (39) ***		0.988		0.980		0.125 (0.102, 0.148)		
Threshold Model	129.360 (45) ***	3.649 (6)	0.989	0.002	0.985	0.005	0.108 (0.087, 0.131)	-0.016	
Metric Model	140.621 (55) ***	11.471 (10)	0.989	0.000	0.988	0.003	0.099 (0.079, 0.119)	-0.010	
Scalar Model	155.021 (65) ***	12.017 (10)	0.989	-0.001	0.989	0.001	0.093 (0.074, 0.112)	-0.006	
Strict Model	181.743 (79) ***	25.795 (14) *	0.987	-0.002	0.990	0.001	0.090 (0.073, 0.108)	-0.003	
* 15-week follow-up*									
Configural Model	32.628 (26) *		0.999		0.998		0.033 (0.000, 0.064)		
Threshold Model	35.270 (28) *	2.446 (2)	0.999	0.000	0.998	0.000	0.033 (0.000, 0.063)	0.000	
Metric Model	36.647 (33) *	1.975 (5)	0.999	0.001	0.999	0.001	0.021 (0.000, 0.054)	-0.011	
Scalar Model	44.677 (38) *	7.982 (5)	0.999	-0.001	0.999	0.000	0.027 (0.000, 0.055)	0.006	
Strict Model	66.584 (45) **	15.934 (7) *	0.996	-0.002	0.997	-0.002	0.045 (0.018, 0.066)	0.018	
**Family income (< 10,000 CNY vs. ≥ 10,000 CNY)**									
*Baseline*									
Configural Model	69.149 (26) ***		0.990		0.983		0.083 (0.060, 0.107)		
Threshold Model	76.096 (31) ***	7.653 (5)	0.989	0.000	0.985	0.002	0.078 (0.056, 0.100)	-0.005	
Metric Model	93.461 (36) ***	4.468 (5)	0.986	-0.003	0.984	-0.001	0.082 (0.062, 0.102)	0.004	
Scalar Model	100.069 (41) ***	5.533 (5)	0.986	0.000	0.985	0.002	0.078 (0.058, 0.097)	-0.004	
Strict Model	122.754 (48) ***	20.351 (7) **	0.982	-0.004	0.984	-0.001	0.081 (0.063, 0.098)	0.003	
* 1-week follow-up*									
Configural Model	112.179 (26) ***		0.989		0.983		0.118 (0.096, 0.140)		
Threshold Model	110.370 (30) ***	2.303 (4)	0.990	0.001	0.986	0.003	0.106 (0.085, 0.127)	-0.012	
Metric Model	113.862 (35) ***	3.011 (5)	0.990	0.000	0.988	0.002	0.097 (0.077, 0.117)	-0.009	
Scalar Model	124.038 (40) ***	8.432 (5)	0.989	-0.001	0.989	0.001	0.094 (0.075, 0.113)	-0.003	
Strict Model	153.245 (47) ***	27.049 (7) ***	0.987	-0.003	0.988	-0.001	0.097 (0.080, 0.115)	0.003	
* 15-week follow-up*									
Configural Model	32.628 (26) *		0.999		0.998		0.033 (0.000, 0.064)		
Threshold Model	35.270 (28) *	2.446 (2)	0.999	0.000	0.998	0.000	0.033 (0.000, 0.063)	0.000	
Metric Model	36.647 (33) *	2.446 (2)	0.999	0.001	0.999	0.001	0.021 (0.000, 0.054)	-0.011	
Scalar Model	44.677 (38) *	7.982 (5)	0.999	-0.001	0.999	0.000	0.027 (0.000, 0.055)	0.006	
Strict Model	66.584 (45) **	15.934 (7) *	0.996	-0.002	0.997	-0.002	0.045 (0.018, 0.066)	0.018	
**Part-time employment (Yes vs. No)**									
*Baseline*									
Configural Model	76.064 (26) ***		0.989		0.982		0.090 (0.067, 0.113)		
Threshold Model	73.674 (31) ***	3.218 (5)	0.991	0.002	0.987	0.005	0.076 (0.054, 0.098)	-0.014	
Metric Model	77.907 (36) ***	6.736 (5)	0.991	0.000	0.989	0.002	0.070 (0.048, 0.091)	-0.006	
Scalar Model	81.348 (41) ***	3.820 (5)	0.991	0.000	0.991	0.002	0.064 (0.043, 0.084)	-0.006	
Strict Model	98.225 (48) ***	13.013 (7)	0.989	-0.002	0.990	-0.001	0.066 (0.047, 0.085)	0.002	
* 1-week follow-up*									
Configural Model	91.441 (26) ***		0.992		0.987		0.103 (0.080, 0.126)		
Threshold Model	86.458 (30) ***	2.566 (4)	0.993	0.001	0.990	0.003	0.089 (0.067, 0.111)	-0.014	
Metric Model	87.845 (35) ***	5.196 (5)	0.993	0.000	0.992	0.002	0.079 (0.059, 0.100)	-0.009	
Scalar Model	93.029 (40) ***	4.803 (5)	0.993	0.000	0.993	0.001	0.074 (0.055, 0.094)	-0.005	
Strict Model	103.967 (47) ***	10.562 (7)	0.993	0.000	0.994	0.001	0.071 (0.053, 0.090)	-0.003	
* 15-week follow-up*									
Configural Model	32.628 (26) *		0.999		0.998		0.033 (0.000, 0.064)		
Threshold Model	35.270 (28) *	2.446 (2)	0.999	0.000	0.998	0.000	0.033 (0.000, 0.063)	0.000	
Metric Model	36.647 (33) *	1.975 (5)	0.999	0.001	0.999	0.001	0.021 (0.000, 0.054)	-0.011	
Scalar Model	44.677 (38) *	7.982 (5)	0.999	-0.001	0.999	0.000	0.027 (0.000, 0.055)	0.006	
Strict Model	66.584 (45) **	15.934 (7) *	0.996	-0.002	0.997	-0.002	0.045 (0.018, 0.066)	0.018	
**Leisure-time sports involvement (Yes vs. No)**									
*Baseline*									
Configural Model	70.149 (26) ***		0.990		0.984		0.084 (0.061, 0.108)		
Threshold Model	82.125 (30) ***	10.212 (4) *	0.988	-0.002	0.983	0.000	0.085 (0.063, 0.107)	0.001	
Metric Model	81.370 (35) ***	2.118 (5)	0.989	0.001	0.987	0.004	0.074 (0.053, 0.096)	-0.011	
Scalar Model	93.929 (40) ***	11.695 (5) *	0.988	-0.002	0.987	0.000	0.075 (0.055, 0.095)	0.001	
Strict Model	131.544 (47) ***	30.673 (7) ***	0.981	-0.007	0.983	-0.004	0.087 (0.069, 0.104)	0.012	
* 1-week follow-up*									
Configural Model	81.444 (26) ***		0.993		0.988		0.094 (0.072, 0.118)		
Threshold Model	87.776 (28) ***	5.499 (2)	0.992	-0.001	0.988	0.000	0.094 (0.072, 0.117)	0.000	
Metric Model	96.528 (33) ***	7.801 (5)	0.992	0.000	0.990	0.001	0.090 (0.069, 0.111)	-0.005	
Scalar Model	100.692 (38) ***	1.918 (5)	0.992	0.000	0.991	0.001	0.083 (0.064, 0.103)	-0.007	
Strict Model	119.248 (45) ***	18.067 (7) *	0.990	-0.001	0.991	0.000	0.083 (0.065, 0.101)	0.000	
* 15-week follow-up*									
Configural Model	32.628 (26) *		0.999		0.998		0.033 (0.000, 0.064)		
Threshold Model	35.270 (28) *	2.446 (2)	0.999	0.000	0.998	0.000	0.033 (0.000, 0.063)	0.000	
Metric Model	36.647 (33) *	1.975 (5)	0.999	0.001	0.999	0.001	0.021 (0.000, 0.054)	-0.011	
Scalar Model	44.677 (38) *	7.982 (5)	0.999	-0.001	0.999	0.000	0.027 (0.000, 0.055)	0.006	
Strict Model	66.584 (45) **	15.934 (7) *	0.996	-0.002	0.997	-0.002	0.045 (0.018, 0.066)	0.018	
Threshold			≥ 0.900	≤ 0.010	≥ 0.900	≤ 0.010	≤ 0.080	≤ 0.015	

The bold type represents the classification. The italics represent the measure time

Abbreviations: *RSES* Rosenberg Self-Esteem Scale, *χ*^*2*^ Chi-square, *df* degrees of freedom, *CFI* comparative fit index, *TLI* Tucker-Lewis index, *RMSEA* root mean square error of approximation, *Δ* a change in *χ*^*2*^, *df*, CFI, TLI, and RMSEA

***P < 0.001

**P < 0.010

*P < 0.050

We also examined the CMI results for the RSES-10 (see Supplementary Material, Table S3) for comparison. The strict model was achieved for both the 1-week follow-up and 15-week follow-up data. But for the baseline data, the academic year, part-time employment, and sports engagement subgroups showed the measurement invariance only in the threshold model.

#### Longitudinal measurement invariance

Table 4 shows the LMI results across the three waves (i.e., baseline, 1-week follow-up, 15-week follow-up) for the RSES-7 and RSES-10. It was found that all the indicators met the criteria, and strict measurement invariance was held for both models, suggesting that our participants’ self-esteem scores remained consistent across the 15 weeks of the study.

**Table 4:** Longitudinal measurement invariances for the RSES-7 across three time points: baseline, 1-week follow-up, and 15-week follow-up

Model	*χ*^*2*^ (*df*)	Δ*χ*^*2*^ (Δ*df*)	CFI	ΔCFI	TLI	ΔTLI	RMSEA (90% CI)	ΔRMSEA
RSES-10								
Configural Model	1413.860 (360) ***		0.966		0.959		0.078 (0.074, 0.082)	
Threshold Model	1413.503 (380) ***	24.427 (20)	0.967	0.001	0.962	0.003	0.075 (0.071, 0.079)	-0.003
Metric Model	1379.322 (396) ***	18.562 (16)	0.968	0.002	0.965	0.003	0.072 (0.068, 0.076)	-0.003
Scalar Model	1409.832 (412) ***	38.861 (16) **	0.968	0.000	0.966	0.001	0.071 (0.067, 0.075)	-0.001
Strict Model	1303.128 (432) ***	75.872 (20) ***	0.972	0.004	0.972	0.006	0.065 (0.061, 0.069)	-0.006
RSES-7								
Configural Model	449.703 (153) ***		0.988		0.983		0.064 (0.057, 0.070)	
Threshold Model	449.507 (167) ***	15.887 (14)	0.988	0.001	0.985	0.002	0.059 (0.053, 0.066)	-0.004
Metric Model	447.994 (177) ***	4.610 (10)	0.989	0.000	0.986	0.001	0.056 (0.050, 0.063)	-0.003
Scalar Model	469.642 (187) ***	28.496 (10) **	0.988	0.000	0.987	0.000	0.056 (0.050, 0.062)	0.000
Strict Model	499.239 (201) ***	59.858 (14) ***	0.987	-0.001	0.987	0.000	0.056 (0.049, 0.062)	-0.001
Threshold			≥ 0.900	≤ 0.010	≥ 0.900	≤ 0.010	≤ 0.080	≤ 0.015

Abbreviations: *RSES* Rosenberg Self-Esteem Scale, *χ*^*2*^ Chi-square, *df* degrees of freedom, *CFI* comparative fit index, *TLI* Tucker-Lewis index, *RMSEA* root mean square error of approximation, *CI* confidence interval, *Δ* a change in *χ*^*2*^, *df*, CFI, TLI, and RMSEA

***P < 0.001

**P < 0.010

### Convergent validity

**Fig. 1:**
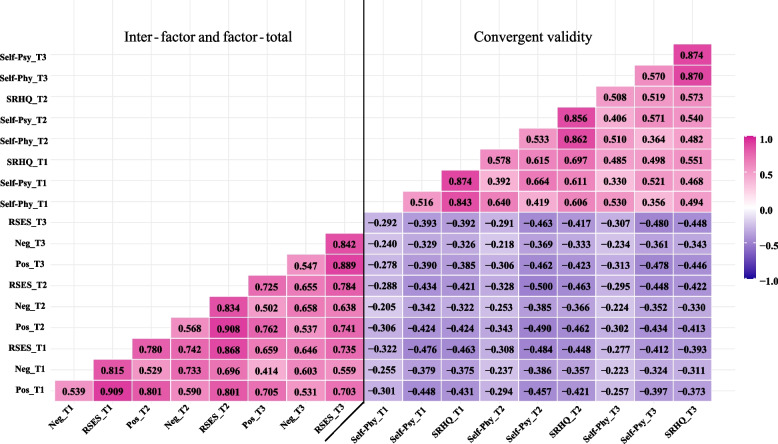
Spearman inter‒factor, factor‒total and convergent validity correlations between the RSES-7 and SRHQ Color gradient represents correlation level. Pink represents a positive correlation. Purple represents a negative correlation Abbreviations: *Pos* positive subscale, *Neg* negative subscale, *RSES* Rosenberg Self-Esteem Scale, *Self-Phy* Self-Rated Physical Condition, *Self-Psy* Self-Rated Psychological Condition, *SRHQ* Self-Rated Health Questionnaire, *T1* baseline, *T2* 1-week follow-up, *T3* 15-week follow-up

The left half of Fig. 1 shows the factor-factor and factor-total score correlations for the RSES-7 (AVE: 0.640‒0.866, CR: 0.784‒0.875, see the Supplementary Material, Table S4, for more details), and the right half shows the correlation between the RSES-7 and SRHQ scores measured at the three waves. The factors of the RSES-7 were positively correlated with each other as well as with the total score. The weakest relationship was observed between the negative factor score measured at baseline and the positive factor score measured at the third wave (*r* = 0.414), while the strongest relationship was found between the positive factor score and the total score of the RSES measured at baseline (*r* = 0.909). In addition, the RSES-7 scores were negatively associated with the SRHQ scores, ranging from -0.205 to -0.500. Similar results were also documented for the RSES-10 (see Supplementary Material, Figure S1, for more details).

### Internal consistency

Cronbach’s α and McDonald’s ω were used to evaluate the internal consistency of the subscales and the total scores of the most recommended form—the RSES-7 for the three waves (see Table 5). The results showed that the RSES-7 had excellent reliability (Cronbach’s α = 0.905‒0.937; McDonald’s ω = 0.904‒0.936), as well as the RSES-10 (Cronbach’s α = 0.911‒0.942; McDonald’s ω = 0.915‒0.944; see Supplementary Material, Table S5, for more details).

**Table 5:** Internal consistency and test–retest reliability: RSES-7 and SRHQ

Variables	RSES-7			SRHQ		
	Global	Positive	Negative	Global	Self-Phy	Self-Psy
Cronbach’s α (95% CI)						
Baseline	0.905 (0.892, 0.918)	0.897 (0.882, 0.911)	—	0.821	—	—
1-week follow-up	0.937 (0.928, 0.946)	0.940 (0.932, 0.949)	—	0.857	—	—
15-week follow-up	0.928 (0.917, 0.938)	0.933 (0.923, 0.942)	—	0.802	—	—
McDonald’s ω (95% CI)						
Baseline	0.904 (0.891, 0.917)	0.897 (0.882, 0.911)	—	—	—	—
1-week follow-up	0.936 (0.927, 0.944)	0.940 (0.931, 0.948)	—	—	—	—
15-week follow-up	0.925 (0.914, 0.935)	0.930 (0.921, 0.940)	—	—	—	—
ICC (95% CI)						
ICC (T1, T2)	0.869 (0.827, 0.899)	0.837 (0.802, 0.866)	0.717 (0.664, 0.762)	0.710 (0.658, 0.754)	0.637 (0.580, 0.688)	0.693 (0.640, 0.739)
ICC (T2, T3)	0.808 (0.774, 0.838)	0.793 (0.757, 0.824)	0.663 (0.609, 0.711)	0.603 (0.543, 0.657)	0.521 (0.453, 0.583)	0.565 (0.501, 0.623)
ICC (T1, T3)	0.752 (0.663, 0.813)	0.743 (0.679, 0.793)	0.579 (0.494, 0.649)	0.565 (0.500, 0.624)	0.545 (0.479, 0.605)	0.512 (0.441, 0.576)
SEM						
SEM (T1, T2)	1.172	0.921	0.718	0.634	0.372	0.404
SEM (T2, T3)	1.426	1.045	0.766	0.693	0.418	0.433
SEM (T1, T3)	1.578	1.131	0.844	0.770	0.430	0.467

This table shows ordinal forms of Cronbach’s α and McDonald’s ω. Standard error of measurement was calculated as “SD × sqrt (1-ICC)”. The McDonald’s ω and the 95% confidential interval of Cronbach’s α cannot be calculated due to the subscales containing only one or two item

Abbreviations: *RSES* Rosenberg Self-Esteem Scale, *SRHQ* Self-Rated Health Questionnaire, *Self-Phy* Self-Rated Physical Condition, *Self-Psy* Self-Rated Psychological Condition, *ICC* Intraclass correlation coefficient, *SEM* Standard error of measurement

### Test–retest reliability

The test–retest reliability of the RSES-7 is reported in Table 5. The overall scale and the positive subscale showed adequate results, but not the negative subscale (ICC = 0.579‒0.717). The RSES-10 also displayed similar results (ICC = 0.642‒0.790), with low test–retest reliability for the negative schedule (see Supplementary Material, Table S5, for more details).

## Discussion

This paper presents a validation of the Chinese version of the Rosenberg Self-Esteem Scale (RSES), using a three-wave assessment to examine its main psychometric properties and measurement invariances. The findings add another piece of robust evidence to support the ongoing psychometric evaluation of the RSES. Given the current context in China and the results of the tests conducted, the RSES-7, which is a modified version of the RSES that excludes Items 5, 8, and 9, has been identified as a potentially more suitable measure for self-esteem. In this study, this brief version, which incorporated simplified Chinese language, demonstrated robust reliability, validity, and measurement invariance.

 Converging evidence demonstrates that response artifacts (e.g., social desirability) may occur when all questions are stated in one direction, and leads to questionable test results [59]. To partially mitigate the potentially invalidating effects of acquiescence, the RSES was designed to consist of five positively worded and five negatively worded items [59]. However, including positive and negative wording to examine the same dimension might lead to response bias, so threatening validity; this is a phenomenon known as the wording effect [60, 61]. Given the specificity of the different cohorts used to examine the properties of the RSES and the inherent differences between Eastern and Western cultures, even when the factor structure is known, it is necessary to perform EFA on the data from different cohorts to further examine the factor loadings and cross-loading phenomena, and identify potential and fundamental issues with the items. Items 5, 8, and 9, all of whichare negatively worded, exhibited inapplicability, and the reason for this was worth exploring. Cross-cultural differences have, therefore, been observed in Chinese versions of the RSES, and a similar situation has been identified in other language versions [21, 22, 24, 62]. A multi-center cross-cultural study involving nearly 17 000 participants from 53 countries found that participants responded truthfully to positively worded items, while showing significant concealment for negatively worded items [9]. This indicates that people from many cultures tend to be biased toward negatively worded items. Additionally, a study across three countries showed that some respondent experience difficulty answering the negatively-worded questions effectively, resulting in serious consequences (e.g., low scale reliability) [63].

The reasons for the inconsistent factor structure regarding Items 5, 8, and 9 are worth exploring. Self-esteem is rooted in Western culture and expresses a greater emphasis on the self as a valuded, independent individual. In China, although there has been a tremendous increase in people’s literacy and self-awareness, humility and altruism are still significant values in Chinese culture. In Eastern cultures, people are more inclined to situate the self in interactions with others, which is an inevitable cultural difference compared to in the West [64]. From an early age, Chinese children are often taught to be humble and that pride makes people fall behind. This may lead to the inconsistent dimensional attribution of Item 5 of the RSES [65]. Sixty-eight percent of the impact of social media use on mental health is mediated by self-esteem [66], and in the Internet era, contacting successful people worldwide has become easier. Over time, this may elicit a sense of falling behind. For example, respondents to the RSES who major in medicine may be exceptional, hard-working, and self-demanding individuals [67], but they might still perceive themselves as a failure compared to their peers, leading to inconsistent dimensional attributions for Item 9. Whether to remove Item 8 has been of long-standing debate among scholars [68]. The discrepant understanding of the word “wish” in different cultural contexts and ideas about modesty in Chinese culture have led to the phenomenon whereby people with high self-esteem may also hope for continued respect [65]. Due to the inevitable cultural differences, to date, there has been no particularly effective solution for Item 8 [69]. However, the present study, which was based on a three-wave design, offers strong evidence for the deletion of Item 8.

Scale maladaptation in cross-cultural applications is the norm. Furthermore, Chinese people are often characterized by dialecticism [70]. This is reflected in a scale that tends to support both sides of the issue, that is, both positive and negative expressions of self-esteem. A cross-cultural study between China and US showed that four of the five negatively-worded items were answered differently by respondents from the two countries [71]. Some cross-cultural studies exclude negatively worded items when using the RSES [62], which is the reason why we explored five models.

Overall, the present study, which utilized a substantial sample across three waves, yielded consistent results that provide compelling evidence for cross-cultural differences regarding Items 5, 8, and 9. When the oblique rotation was applied, the pattern load, which is essentially a regression coefficient, exceeded 1. Consequently, the RSES-7 was considered to be the best model even when the factor loading for Item 6 was greater than 1. Although less information is inevitably collected when items are deleted, when we removed items from the negatively-worded dimension, we retained the two-factor structure. Generally, the RSES-7 is an easy-to-use instrument with strong validity data for self-esteem measurement.

Self-esteem varies widely across groups, and a large study based on a sample of nearly one million participants found an age-related increase in self-esteem from late adolescence to mid-adulthood, and that self-esteem was significantly higher in men than in women [47]. Group comparisons and longitudinal changes are fundamental to understanding the role of self-esteem in psychological well-being. Therefore, it is important to examine whether the measurement properties of the RSES are comparable across groups (CMI) and stable across time (LMI). However, few studies have tested these forms of measurement invariance for the RSES. With our CMI evidence, we found that subgroups of students who participate in sports, have higher family incomes, and are involved in part-time jobs, have higher self-esteem [72]. With all eight subgroups, the RSES-7 achieved strict invariance across the three waves, which means that differences in self-esteem itself are well-identified when comparing these subgroups.

Based on a three-wave design, the RSES-7 achieved the strict invariance models in longitudinal CFA, indicating that the residual invariance constrains factor loadings, item intercepts, and residual variances, and does not change across time points. This implies that if the scores had changed over time, this would have been caused by a change in the latent variable and not by a change in item understanding. The present study adds LMI across 15 weeks to the psychometric evidence for the RSES; the LMI provided robust evidence regarding the assessed construct and had the same meaning across time points, which will support the design of for future longitudinal studies.

### Recommendations

The RSES-10 has a suboptimal factor structure, validity, and measurement invariance, yet it is advantageous for cross-cultural comparisons; the RSES-7 is the simplest and most robust form of the RSES and has adequate psychometric properties and measurement invariance; therefore, we recommend the RSES-7 as the preferred solution for use with Chinese university students.

### Strengths and weaknesses

This paper presents a large-scale validation of the Chinese Macau adaptation of the RSES in the Chinese mainland. After a dramatic change in the Chinese socio-cultural context, the study re-evaluated the psychometric properties of the previously translated traditional language version of the RSES by utilizing the simplified Chinese language. Ultimately, a more concise and potentially applicable form of the RSES—a 7-item form—was proposed. Second, by retaining the two original factors with a reduced number of items, the RSES-7 has the potential to alleviate the response burden on respondents. Third, although the RSES has been validated worldwide, the longitudinal design used here (baseline, 1-week follow-up, 15-week follow-up), with a large sample size, was a particular advantage and provided robust evidence. Lastly, a comprehensive and systematic assessment of the psychometric properties based on COSMIN and STROBE guidelines, in which CMI was evaluated for a wide range of socio-demographic variables and LMI was estimated for the three-wave measure, was unprecedented.

Nonetheless, some limitations of our study need to be considered. The respondents were drawn from one university, representing a specific group of Chinese millennials in the medical specialty. The homogeneity of the population was taken to provide a more accurate historical and social focus but it limits the generalizability of the findings to the same age groups. In the same vein, the present study tested the RSES-7 in the Chinese mainland context and hence, its usability in other cultural contexts remains to be explored. Third, although item removal was accomplished while retaining a two-factor structure, reduced information resulting from the use of fewer items is inevitable. Finally, although it is noteworthy that we used the original 10-item RSES to retrieve the data from which the seven item selected RSES-7 were identified, the findings of participants’ responses may still have been confounded by removing three items. As a result, the psychometric qualities of the RSES-7 require further examination.

### Future directions

Further investigation is warranted through a comprehensive survey of healthcare students from diverse regions and specialties to determine if the aforementioned findings can be replicated. In addition, as a more concise version, the RSES-7 requires comparative analysis with other self-esteem scales to further assess its psychometric properties. In response to the item deletions, while we tentatively conclude that they were not due to methodological effects, the underlying linguistic reasons need to be further explored. Lastly, the RSES is available in many languages, but large-scale cross-cultural measurement invariance has not been evaluated. In the future, we hope to join forces with researchers from other countries and regions to further explore the cross-cultural invariance of the RSES.

## Conclusion

This study revealed that Items 5, 8, and 9 of the RSES pose potential risks to its structural stability and may hinder cross-cultural comparability. These findings enhance our understanding of the RSES. Cross-sectional measurement invariance across eight subgroups, and longitudinal measurement invariance based on three-wave assessments, were well demonstrated, providing support for the psychometric qualities of the RSES-7. This enlightens future studies to validate the RSES-7 in different regions and populations. If its psychometric properties remain adequate, this simplified form of the RSES would facilitate a lower response burden, more efficient analysis, and wider application.

### Supplementary Information

**Additional file 1: Fig. S1.** Spearman inter‒factor, factor‒total, and convergent validity correlations between the RSES-10 and SRHQ. **Table S1.** Characteristics of participants (*N* = 481). **Table S2.** EFA factor loadings: RSES-9, RSES-8, RSES-6, and RSES-5. **Table S3.** Cross-sectional measurement invariances: RSES-10 with two factors. **Table S4.** The average variance extracted and construct reliability of the RSES-7 in convergent validity. **Table S5.** Internal consistency and test–retest reliability: RSES-10 and SRHQ.

## Data Availability

The data generated or analyzed during this study are not publicly available due to restrictions imposed by the ethics committee. The dataset supporting the conclusions is available upon reasonable request to the last author.

## Abbreviations

CFA: confirmatory factor analysis
CFI: comparative fit index
CMI: cross-sectional measurement invariance
COSMIN: COnsensus-based Standards for the selection of health Measurement INstruments
EFA: exploratory factor analysis
ICC: intraclass correlation coefficient
KMO: Kaiser–Meyer–Olkin
LMI: longitudinal measurement invariance
RMSEA: root mean square error of approximation
RSES: Rosenberg Self-Esteem Scale
SRHQ: Self-Rated Health Questionnaire
SRMR: standardized root mean residual
STROBE: STrengthening the Reporting of OBservational studies in Epidemiology
TLI: Tucker-Lewis index
WLSMV: weighted least squares mean and variance adjusted
