# Supplementary material for: Validation of the Chinese version of the Rosenberg Self-Esteem Scale: evidence from a three-wave longitudinal study
Source: BMC Psychol. 2023 Oct 18;11:345. doi: 10.1186/s40359-023-01293-1 (PMC10585735; doi:10.1186/s40359-023-01293-1)
Supplement: Supplementary file 1 — Additional file 1: Fig. S1. Spearman inter‒factor, factor‒total, and convergent validity correlations between the RSES-10 and SRHQ. Table S1. Characteristics of participants (N = 481). Table S2. EFA factor loadings: RSES-9, RSES-8, RSES-6, and RSES-5. Table S3. Cross-sectional measurement invariances: RSES-10 with two factors. Table S4. The average variance extracted and construct reliability of the RSES-7 in convergent validity. Table S5. Internal consistency and test–retest reliability: RSES-10 and SRHQ. [file 40359_2023_1293_MOESM1_ESM.docx]

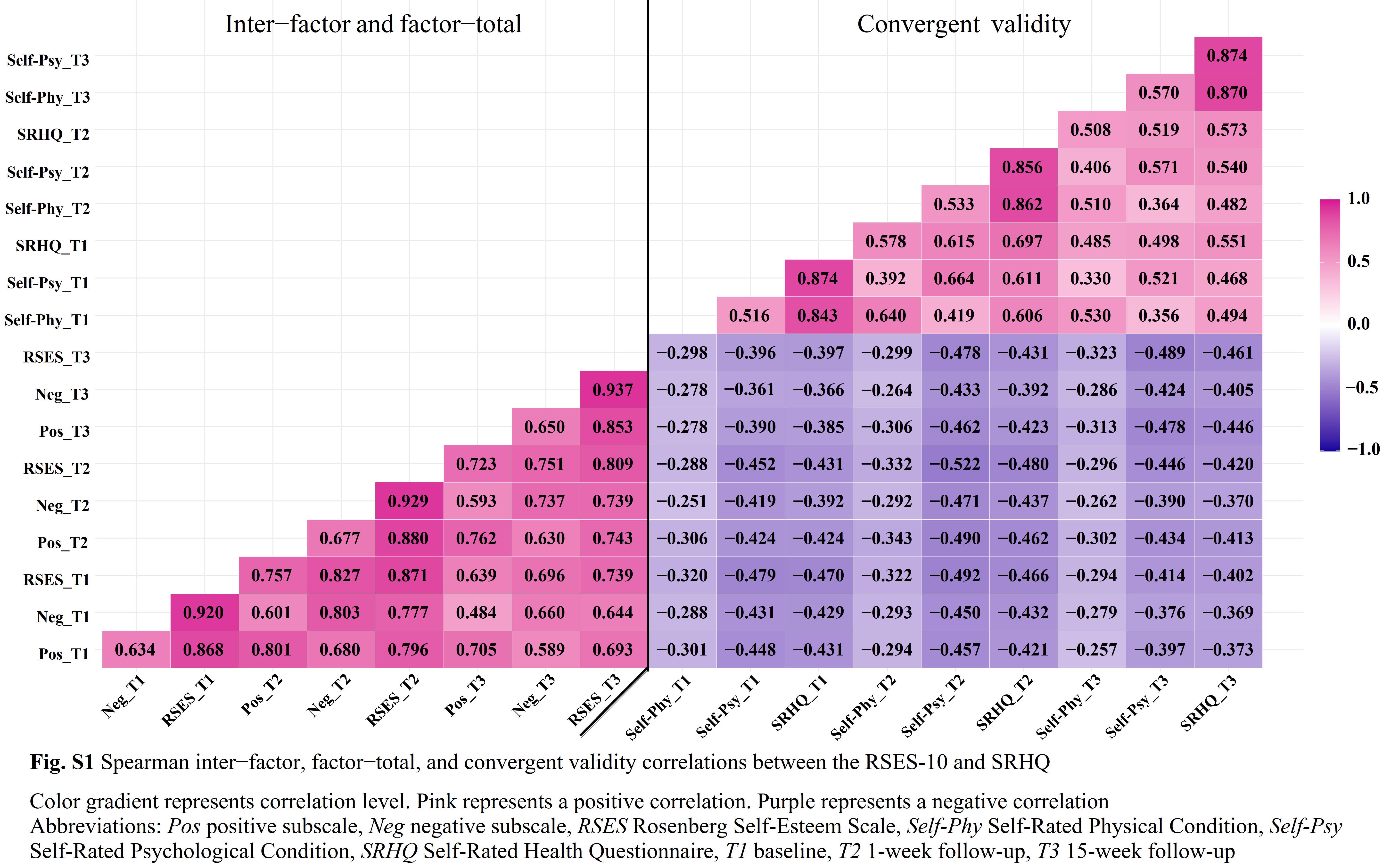


**Table S1** Characteristics of participants (*N* = 481)

| Variable | *N* (%) | RSES-10 mean scores (SD) | | |
| --- | --- | --- | --- | --- |
|  |  | Baseline | 1-week follow-up | 15-week follow-up |
| Gender |  |  |  |  |
| Male | 112 (23.285) | 27.902 (5.013) | 28.089 (5.248) | 28.580 (4.634) |
| Female | 369 (76.715) | 26.978 (4.307) | 27.667 (4.340) | 28.184 (4.357) |
| Age |  |  |  |  |
| < 20 | 214 (44.491) | 27.136 (4.669) | 27.607 (4.535) | 28.084 (4.392) |
| ≥ 20 | 267 (55.509) | 27.240 (4.355) | 27.891 (4.594) | 28.431 (4.447) |
| Home location |  |  |  |  |
| Urban | 173 (35.967) | 27.699 (4.514) | 28.098 (4.571) | 28.601 (4.536) |
| Rural | 182 (37.838) | 27.231 (4.569) | 27.758 (4.840) | 28.495 (4.509) |
| Suburban | 126 (26.195) | 26.444 (4.281) | 27.317 (4.125) | 27.516 (4.065) |
| Single-child status |  |  |  |  |
| Yes | 187 (38.877) | 27.711 (4.505) | 28.160 (4.575) | 28.674 (4.859) |
| No | 294 (61.123) | 26.864 (4.462) | 27.514 (4.549) | 28.024 (4.108) |
| Academic year |  |  |  |  |
| First year | 188 (39.085) | 27.511 (4.505) | 28.069 (4.676) | 28.574 (4.532) |
| Second year | 129 (26.819) | 26.798 (4.916) | 27.380 (4.537) | 27.860 (4.431) |
| Third year | 164 (34.096) | 27.140 (4.116) | 27.720 (4.462) | 28.262 (4.284) |
| Family income | |  |  |  |
| < 10,000 CNY | 215 (44.699) | 26.344 (4.577) | 27.028 (4.511) | 27.540 (4.535) |
| ≥ 10,000 CNY | 266 (55.301) | 27.880 (4.312) | 28.361 (4.531) | 28.872 (4.243) |
| Part-time employment | |  |  |  |
| Yes | 84 (17.464) | 25.750 (4.379) | 26.524 (4.255) | 27.095 (4.414) |
| No | 397 (82.536) | 27.499 (4.462) | 28.028 (4.590) | 28.526 (4.388) |
| Leisure-time sports involvement | | |  |  |
| Yes | 236 (49.064) | 27.530 (4.589) | 27.979 (4.642) | 28.441 (4.416) |
| No | 245 (50.936) | 26.869 (4.383) | 27.559 (4.491) | 28.118 (4.431) |

1 CNY ≈ 0.140 USD

*Abbreviations: RSES* Rosenberg Self-Esteem Scale, *SD* standard deviation

**Table S2** EFA factor loadings: RSES-9, RSES-8, RSES-6, and RSES-5

| Variable | Positive | Negative | KMO test | Bartlett’s test | Cumulative variance |
| --- | --- | --- | --- | --- | --- |
| RSES-9 |  |  | 0.898 | 1920.968 (36) *** | 0.542 |
| RSES01 | 0.709 | -0.031 |  |  |  |
| RSES02 | -0.047 | 0.861 |  |  |  |
| RSES03 | 0.705 | 0.029 |  |  |  |
| RSES04 | 0.816 | -0.141 |  |  |  |
| **RSES05** | **0.428** | **0.276** |  |  |  |
| RSES06 | -0.031 | 0.853 |  |  |  |
| RSES07 | 0.790 | -0.083 |  |  |  |
| **RSES09** | **0.501** | **0.296** |  |  |  |
| RSES10 | 0.594 | 0.117 |  |  |  |
| RSES-7 |  |  | 0.878 | 1679.087 (28) *** | 0.557 |
| RSES01 | 0.701 | -0.011 |  |  |  |
| RSES02 | -0.038 | 0.870 |  |  |  |
| RSES03 | 0.683 | 0.044 |  |  |  |
| RSES04 | 0.798 | -0.113 |  |  |  |
| RSES06 | 0.013 | 0.809 |  |  |  |
| RSES07 | 0.776 | -0.063 |  |  |  |
| **RSES09** | **0.501** | **0.300** |  |  |  |
| RSES-6 |  |  | 0.867 | 974.663 (15) ** | 0.468 |
| RSES01 | 0.665 | 0.039 |  |  |  |
| RSES02 | -0.020 | 0.867 |  |  |  |
| RSES03 | 0.663 | 0.086 |  |  |  |
| RSES04 | 0.769 | -0.055 |  |  |  |
| RSES07 | 0.795 | -0.079 |  |  |  |
| RSES10 | 0.586 | 0.094 |  |  |  |
| RSES-5 |  |  | 0.838 | 827.0874 (10) ** | 0.500 |
| RSES01 | 0.696 |  |  |  |  |
| RSES03 | 0.717 |  |  |  |  |
| RSES04 | 0.730 |  |  |  |  |
| RSES07 | 0.740 |  |  |  |  |
| RSES10 | 0.649 |  |  |  |  |

Bold font indicates items with cross-loading

*Abbreviations:* *RSES* Rosenberg Self-Esteem Scale, *KMO test* Kaiser-Meyer-Olkin test

****P* < 0.001

***P* < 0.010

**Table S3** Cross-sectional measurement invariances: RSES-10 with two factors

| Hypothesis | *χ^2^* (*df*) | Δ*χ^2^* (Δ*df*) | CFI | ΔCFI | TLI | ΔTLI | RMSEA | ΔRMSEA |
| --- | --- | --- | --- | --- | --- | --- | --- | --- |
| **Gender (Male vs. Female)** | | | | | | | | |
| *Baseline* | | | | | | | | |
| Configural Model | 271.372 (68) *** |  | 0.953 |  | 0.938 |  | 0.112 (0.098, 0.126) |  |
| Threshold Model | 269.371 (74) *** | 5.661 (6) | 0.955 | 0.002 | 0.945 | 0.007 | 0.105 (0.092, 0.119) | -0.007 |
| Metric Model | 309.762 (82) *** | 28.927 (8) *** | 0.947 | -0.008 | 0.942 | -0.003 | 0.108 (0.095, 0.121) | 0.003 |
| Scalar Model | 332.596 (90) *** | 18.776 (8) * | 0.944 | -0.003 | 0.944 | 0.002 | 0.106 (0.094, 0.118) | -0.002 |
| Strict Model | 349.304 (100) *** | 25.161 (10) ** | 0.942 | -0.002 | 0.948 | 0.004 | 0.102 (0.090, 0.114) | -0.004 |
| *1-week follow-up* | | | | | | | | |
| Configural Model | 298.123 (68) *** |  | 0.969 |  | 0.959 |  | 0.119 (0.105, 0.133) |  |
| Threshold Model | 281.793 (75) *** | 6.946 (7) | 0.972 | 0.003 | 0.967 | 0.008 | 0.107 (0.094, 0.121) | -0.012 |
| Metric Model | 279.686 (83) *** | 11.343 (8) | 0.974 | 0.001 | 0.972 | 0.005 | 0.099 (0.087, 0.112) | -0.008 |
| Scalar Model | 293.554 (91) *** | 12.078 (8) | 0.973 | -0.001 | 0.973 | 0.002 | 0.096 (0.084, 0.109) | -0.003 |
| Strict Model | 283.146 (101) *** | 13.798 (10) | 0.976 | 0.003 | 0.978 | 0.005 | 0.087 (0.075, 0.099) | -0.010 |
| *15-week follow-up* | | | | | | | | |
| Configural Model | 316.411 (68) *** |  | 0.972 |  | 0.963 |  | 0.124 (0.110, 0.137) |  |
| Threshold Model | 301.827 (76) *** | 5.969 (8) | 0.975 | 0.003 | 0.970 | 0.007 | 0.111 (0.098, 0.125) | -0.012 |
| Metric Model | 283.034 (84) *** | 8.444 (8) | 0.978 | 0.003 | 0.976 | 0.006 | 0.099 (0.087, 0.112) | -0.012 |
| Scalar Model | 295.922 (92) *** | 13.116 (8) | 0.977 | -0.001 | 0.978 | 0.002 | 0.096 (0.084, 0.109) | -0.003 |
| Strict Model | 313.908 (102) *** | 28.114 (10) ** | 0.976 | -0.001 | 0.979 | 0.001 | 0.093 (0.082, 0.105) | -0.003 |
| **Age (< 20 vs. ≥ 20)** | | | | | | | | |
| *Baseline* | | | | | | | | |
| Configural Model | 286.440 (68) *** |  | 0.948 |  | 0.931 |  | 0.116 (0.103, 0.131) |  |
| Threshold Model | 288.479 (74) *** | 7.351 (6) | 0.949 | 0.001 | 0.938 | 0.007 | 0.111 (0.097, 0.124) | -0.006 |
| Metric Model | 270.409 (82) *** | 12.295 (8) | 0.955 | 0.006 | 0.951 | 0.013 | 0.098 (0.086, 0.112) | -0.012 |
| Scalar Model | 296.674 (90) *** | 25.398 (8) ** | 0.951 | -0.004 | 0.951 | 0.000 | 0.098 (0.086, 0.111) | 0.000 |
| Strict Model | 324.969 (100) *** | 34.531 (10) *** | 0.947 | -0.004 | 0.952 | 0.001 | 0.097 (0.086, 0.109) | -0.001 |
| *1-week follow-up* | | | | | | | | |
| Configural Model | 314.835 (68) *** |  | 0.966 |  | 0.955 |  | 0.124 (0.110, 0.138) |  |
| Threshold Model | 300.773 (75) *** | 7.080 (7) | 0.969 | 0.003 | 0.963 | 0.008 | 0.113 (0.100, 0.126) | -0.011 |
| Metric Model | 291.441 (83) *** | 10.859 (8) | 0.971 | 0.002 | 0.969 | 0.006 | 0.103 (0.090, 0.116) | -0.010 |
| Scalar Model | 310.953 (91) *** | 18.755 (8) * | 0.97 | -0.002 | 0.970 | 0.001 | 0.101 (0.089, 0.113) | -0.002 |
| Strict Model | 333.210 (101) *** | 33.015 (10) *** | 0.968 | -0.002 | 0.971 | 0.001 | 0.098 (0.087, 0.110) | -0.002 |
| *15-week follow-up* | | | | | | | | |
| Configural Model | 343.593 (68) *** |  | 0.970 |  | 0.96 |  | 0.131 (0.117, 0.145) |  |
| Threshold Model | 336.685 (76) *** | 10.078 (8) | 0.972 | 0.002 | 0.967 | 0.006 | 0.120 (0.107, 0.134) | -0.010 |
| Metric Model | 323.931 (84) *** | 13.770 (8) | 0.974 | 0.002 | 0.972 | 0.006 | 0.110 (0.097, 0.123) | -0.011 |
| Scalar Model | 328.523 (92) *** | 3.762 (8) | 0.974 | 0.000 | 0.975 | 0.003 | 0.104 (0.092, 0.116) | -0.006 |
| Strict Model | 325.452 (102) *** | 20.496 (10) * | 0.976 | 0.001 | 0.979 | 0.004 | 0.096 (0.085, 0.108) | -0.008 |
| **Home location (Urban vs. Rural vs. Suburban)** | | | | | | | | |
| *Baseline* | | | | | | | | |
| Configural Model | 252.391 (68) *** |  | 0.957 |  | 0.943 |  | 0.106 (0.093, 0.121) |  |
| Threshold Model | 252.683 (74) *** | 7.320 (6) | 0.958 | 0.001 | 0.949 | 0.006 | 0.100 (0.087, 0.114) | -0.006 |
| Metric Model | 262.453 (82) *** | 7.320 (6) | 0.958 | 0.000 | 0.954 | 0.004 | 0.096 (0.083, 0.109) | -0.005 |
| Scalar Model | 281.736 (90) *** | 18.323 (8) * | 0.955 | -0.003 | 0.955 | 0.001 | 0.094 (0.082, 0.107) | -0.002 |
| Strict Model | 307.762 (100) *** | 30.551 (10) ** | 0.952 | -0.004 | 0.957 | 0.001 | 0.093 (0.081, 0.105) | -0.001 |
| *1-week follow-up* | | | | | | | | |
| Configural Model | 291.304 (68) *** |  | 0.968 |  | 0.958 |  | 0.117 (0.103, 0.131) |  |
| Threshold Model | 281.664 (73) *** | 3.072 (5) | 0.970 | 0.002 | 0.963 | 0.005 | 0.109 (0.096, 0.123) | -0.008 |
| Metric Model | 272.361 (81) *** | 11.196 (8) | 0.973 | 0.002 | 0.97 | 0.006 | 0.099 (0.086, 0.112) | -0.010 |
| Scalar Model | 278.319 (89) *** | 5.015 (8) | 0.973 | 0.000 | 0.973 | 0.003 | 0.094 (0.082, 0.107) | -0.005 |
| Strict Model | 279.932 (99) *** | 17.264 (10) | 0.974 | 0.001 | 0.977 | 0.004 | 0.087 (0.075, 0.100) | -0.007 |
| *15-week follow-up* | | | | | | | | |
| Configural Model | 305.527 (68) *** |  | 0.974 |  | 0.966 |  | 0.121 (0.107, 0.135) |  |
| Threshold Model | 301.319 (76) *** | 9.160 (8) | 0.976 | 0.001 | 0.971 | 0.005 | 0.111 (0.098, 0.125) | -0.010 |
| Metric Model | 321.880 (84) *** | 9.160 (8) | 0.974 | -0.001 | 0.973 | 0.001 | 0.109 (0.096, 0.121) | -0.003 |
| Scalar Model | 328.679 (92) *** | 7.570 (8) | 0.974 | 0.000 | 0.975 | 0.003 | 0.104 (0.092, 0.116) | -0.005 |
| Strict Model | 310.444 (102) *** | 13.957 (10) | 0.978 | 0.003 | 0.980 | 0.005 | 0.092 (0.081, 0.104) | -0.011 |
| **Single-child status (Yes vs. No)** | | | | | | | | |
| *Baseline* | | | | | | | | |
| Configural Model | 297.191 (68) *** |  | 0.946 |  | 0.929 |  | 0.119 (0.105, 0.133) |  |
| Threshold Model | 295.579 (74) *** | 7.250 (6) | 0.948 | 0.002 | 0.937 | 0.008 | 0.112 (0.099, 0.125) | -0.007 |
| Metric Model | 271.057 (82) *** | 7.649 (8) | 0.956 | 0.008 | 0.951 | 0.015 | 0.098 (0.085, 0.111) | -0.014 |
| Scalar Model | 278.023 (90) *** | 7.125 (8) | 0.956 | 0.000 | 0.956 | 0.005 | 0.093 (0.081, 0.106) | -0.005 |
| Strict Model | 284.793 (100) *** | 16.188 (10) | 0.957 | 0.001 | 0.961 | 0.005 | 0.088 (0.076, 0.100) | -0.006 |
| *1-week follow-up* | | | | | | | | |
| Configural Model | 298.789 (68) *** |  | 0.968 |  | 0.958 |  | 0.119 (0.105, 0.133) |  |
| Threshold Model | 293.080 (73) *** | 4.445 (5) | 0.970 | 0.001 | 0.962 | 0.005 | 0.112 (0.099, 0.126) | -0.007 |
| Metric Model | 271.035 (81) *** | 5.505 (8) | 0.974 | 0.004 | 0.971 | 0.008 | 0.099 (0.086, 0.112) | -0.013 |
| Scalar Model | 275.012 (89) *** | 2.950 (8) | 0.974 | 0.001 | 0.974 | 0.003 | 0.093 (0.081, 0.106) | -0.006 |
| Strict Model | 281.891 (99) *** | 19.096 (10) * | 0.975 | 0.000 | 0.977 | 0.003 | 0.088 (0.076, 0.100) | -0.006 |
| *15-week follow-up* | | | | | | | | |
| Configural Model | 318.071 (68) *** |  | 0.973 |  | 0.964 |  | 0.124 (0.110, 0.138) |  |
| Threshold Model | 303.951 (76) *** | 5.326 (8) | 0.976 | 0.002 | 0.971 | 0.007 | 0.112 (0.099, 0.125) | -0.012 |
| Metric Model | 286.756 (84) *** | 7.596 (8) | 0.978 | 0.003 | 0.977 | 0.006 | 0.100 (0.088, 0.113) | -0.012 |
| Scalar Model | 291.015 (92) *** | 4.745 (8) | 0.979 | 0.000 | 0.979 | 0.002 | 0.095 (0.083, 0.107) | -0.005 |
| Strict Model | 290.157 (102) *** | 18.865 (10) * | 0.980 | 0.001 | 0.982 | 0.003 | 0.088 (0.076, 0.100) | -0.007 |
| **Academic year (First year vs. Second year vs. Third year)** | | | | | | | | |
| *Baseline* | | | | | | | | |
| Configural Model | 306.854 (102) *** |  | 0.951 |  | 0.936 |  | 0.112 (0.098, 0.127) |  |
| Threshold Model | 312.373 (112) *** | 11.189 (10) | 0.952 | 0.001 | 0.943 | 0.007 | 0.106 (0.092, 0.120) | -0.006 |
| Metric Model | 296.671 (128) *** | 19.696 (16) | 0.960 | 0.008 | 0.958 | 0.015 | 0.091 (0.077, 0.105) | -0.015 |
| Scalar Model | 326.751 (144) *** | 26.734 (16) * | 0.957 | -0.003 | 0.959 | 0.002 | 0.089 (0.076, 0.102) | -0.002 |
| Strict Model | 389.747 (164) *** | 54.125 (20) *** | 0.946 | -0.010 | 0.956 | -0.003 | 0.093 (0.081, 0.105) | 0.004 |
| *1-week follow-up* | | | | | | | | |
| Configural Model | 356.416 (102) *** |  | 0.963 |  | 0.952 |  | 0.125 (0.111, 0.139) |  |
| Threshold Model | 346.078 (110) *** | 5.456 (8) | 0.966 | 0.003 | 0.958 | 0.007 | 0.116 (0.102, 0.130) | -0.009 |
| Metric Model | 354.544 (126) *** | 27.434 (16) * | 0.967 | 0.001 | 0.965 | 0.006 | 0.107 (0.094, 0.120) | -0.009 |
| Scalar Model | 389.131 (142) *** | 29.747 (16) * | 0.964 | -0.003 | 0.966 | 0.001 | 0.105 (0.092, 0.117) | -0.002 |
| Strict Model | 407.870 (162) *** | 35.793 (20) * | 0.965 | 0.000 | 0.971 | 0.004 | 0.098 (0.086, 0.109) | -0.007 |
| *15-week follow-up* | | | | | | | | |
| Configural Model | 443.740 (102) *** |  | 0.989 |  | 0.985 |  | 0.145 (0.131, 0.159) |  |
| Threshold Model | 442.475 (116) *** | 10.404 (14) | 0.989 | 0.000 | 0.988 | 0.002 | 0.133 (0.120, 0.146) | -0.012 |
| Metric Model | 434.377 (132) *** | 18.343 (16) | 0.990 | 0.001 | 0.99 | 0.002 | 0.120 (0.107, 0.133) | -0.013 |
| Scalar Model | 456.466 (148) *** | 17.407 (16) | 0.990 | 0.000 | 0.991 | 0.001 | 0.114 (0.103, 0.126) | -0.006 |
| Strict Model | 466.672 (168) *** | 31.833 (20) * | 0.990 | 0.000 | 0.992 | 0.001 | 0.106 (0.094, 0.117) | -0.009 |
| **Family income (< 10,000 CNY vs. ≥ 10,000 CNY)** | | | | | | | | |
| *Baseline* | | | | | | | | |
| Configural Model | 280.387 (68) *** |  | 0.949 |  | 0.932 |  | 0.114 (0.100, 0.128) |  |
| Threshold Model | 283.267 (74) *** | 8.170 (6) | 0.950 | 0.001 | 0.939 | 0.006 | 0.109 (0.095, 0.122) | -0.006 |
| Metric Model | 270.689 (82) *** | 7.320 (6) | 0.955 | 0.005 | 0.950 | 0.011 | 0.098 (0.085, 0.111) | -0.011 |
| Scalar Model | 279.589 (90) *** | 9.222 (8) | 0.954 | 0.000 | 0.954 | 0.004 | 0.094 (0.081, 0.106) | -0.004 |
| Strict Model | 293.346 (100) *** | 23.729 (10) ** | 0.953 | -0.001 | 0.958 | 0.004 | 0.090 (0.078, 0.102) | -0.004 |
| *1-week follow-up* | | | | | | | | |
| Configural Model | 311.101 (68) *** |  | 0.965 |  | 0.954 |  | 0.122 (0.109, 0.136) |  |
| Threshold Model | 304.184 (73) *** | 2.648 (5) | 0.967 | 0.002 | 0.959 | 0.005 | 0.115 (0.102, 0.129) | -0.007 |
| Metric Model | 292.753 (81) *** | 10.077 (8) | 0.970 | 0.003 | 0.966 | 0.007 | 0.104 (0.092, 0.118) | -0.011 |
| Scalar Model | 304.083 (89) *** | 9.394 (8) | 0.969 | 0.000 | 0.969 | 0.003 | 0.100 (0.088, 0.113) | -0.004 |
| Strict Model | 345.151 (99) *** | 45.067 (10) *** | 0.965 | -0.004 | 0.968 | -0.001 | 0.102 (0.090, 0.114) | 0.001 |
| *15-week follow-up* | | | | | | | | |
| Configural Model | 351.479 (68) *** |  | 0.969 |  | 0.959 |  | 0.132 (0.118, 0.146) |  |
| Threshold Model | 333.796 (76) *** | 7.048 (8) | 0.972 | 0.003 | 0.967 | 0.008 | 0.119 (0.106, 0.132) | -0.013 |
| Metric Model | 314.389 (84) *** | 9.160 (8) | 0.975 | 0.003 | 0.973 | 0.006 | 0.107 (0.095, 0.120) | -0.012 |
| Scalar Model | 320.644 (92) *** | 4.359 (8) | 0.975 | 0.000 | 0.976 | 0.003 | 0.102 (0.090, 0.114) | -0.005 |
| Strict Model | 320.755 (102) *** | 21.414 (10) * | 0.976 | 0.001 | 0.979 | 0.003 | 0.095 (0.083, 0.106) | -0.007 |
| **Part-time employment (Yes vs. No)** | | | | | | | | |
| *Baseline* | | | | | | | | |
| Configural Model | 270.239 (68) *** |  | 0.954 |  | 0.939 |  | 0.111 (0.098, 0.126) |  |
| Threshold Model | 264.433 (74) *** | 3.934 (6) | 0.957 | 0.003 | 0.948 | 0.008 | 0.104 (0.090, 0.117) | -0.008 |
| Metric Model | 230.263 (82) *** | 7.157 (8) | 0.966 | 0.010 | 0.963 | 0.016 | 0.087 (0.074, 0.100) | -0.017 |
| Scalar Model | 230.848 (90) *** | 5.916 (8) | 0.968 | 0.002 | 0.968 | 0.005 | 0.081 (0.068, 0.094) | -0.006 |
| Strict Model | 241.098 (100) *** | 15.608 (10) | 0.968 | 0.000 | 0.971 | 0.003 | 0.077 (0.064, 0.089) | -0.004 |
| *1-week follow-up* | | | | | | | | |
| Configural Model | 258.389 (68) *** |  | 0.974 |  | 0.965 |  | 0.108 (0.094, 0.122) |  |
| Threshold Model | 247.960 (73) *** | 3.113 (5) | 0.976 | 0.002 | 0.970 | 0.005 | 0.100 (0.087, 0.114) | -0.008 |
| Metric Model | 253.879 (81) *** | 15.556 (8) * | 0.976 | 0.000 | 0.974 | 0.003 | 0.094 (0.081, 0.108) | -0.006 |
| Scalar Model | 259.118 (89) *** | 6.636 (8) | 0.977 | 0.000 | 0.976 | 0.003 | 0.089 (0.077, 0.102) | -0.005 |
| Strict Model | 259.804 (99) *** | 15.475 (10) | 0.978 | 0.001 | 0.980 | 0.004 | 0.082 (0.070, 0.095) | -0.007 |
| *15-week follow-up* | | | | | | | | |
| Configural Model | 287.432 (68) *** |  | 0.977 |  | 0.969 |  | 0.116 (0.102, 0.130) |  |
| Threshold Model | 271.439 (75) *** | 4.737 (7) | 0.979 | 0.002 | 0.975 | 0.006 | 0.105 (0.091, 0.118) | -0.012 |
| Metric Model | 270.255 (83) *** | 10.374 (8) | 0.980 | 0.001 | 0.978 | 0.003 | 0.097 (0.084, 0.110) | -0.008 |
| Scalar Model | 275.438 (91) *** | 7.003 (8) | 0.980 | 0.000 | 0.981 | 0.002 | 0.092 (0.080, 0.105) | -0.005 |
| Strict Model | 281.624 (101) *** | 18.576 (10) * | 0.981 | 0.000 | 0.983 | 0.002 | 0.086 (0.075, 0.098) | -0.006 |
| **Leisure-time sports involvement (Yes vs. No)** | | | | | | | | |
| *Baseline* | | | | | | | | |
| Configural Model | 295.947 (68) *** |  | 0.947 |  | 0.930 |  | 0.118 (0.105, 0.132) |  |
| Threshold Model | 312.193 (73) *** | 15.070 (5) * | 0.944 | -0.003 | 0.931 | 0.002 | 0.117 (0.104, 0.130) | -0.001 |
| Metric Model | 273.436 (81) *** | 2.751 (8) | 0.955 | 0.011 | 0.950 | 0.019 | 0.100 (0.087, 0.113) | -0.017 |
| Scalar Model | 287.172 (89) *** | 13.034 (8) | 0.954 | -0.001 | 0.953 | 0.003 | 0.096 (0.084, 0.109) | -0.003 |
| Strict Model | 310.631 (99) *** | 30.221 (10) ** | 0.951 | -0.003 | 0.955 | 0.002 | 0.094 (0.083, 0.106) | -0.002 |
| *1-week follow-up* | | | | | | | | |
| Configural Model | 270.326 (68) *** |  | 0.970 |  | 0.960 |  | 0.111 (0.098, 0.126) |  |
| Threshold Model | 275.742 (71) *** | 6.239 (3) | 0.970 | 0.000 | 0.962 | 0.001 | 0.110 (0.096, 0.124) | -0.002 |
| Metric Model | 257.800 (79) *** | 6.330 (8) | 0.973 | 0.004 | 0.970 | 0.008 | 0.097 (0.084, 0.111) | -0.013 |
| Scalar Model | 261.825 (87) *** | 2.524 (8) | 0.974 | 0.001 | 0.973 | 0.003 | 0.092 (0.079, 0.104) | -0.006 |
| Strict Model | 262.044 (97) *** | 16.128 (10) | 0.976 | 0.001 | 0.977 | 0.004 | 0.084 (0.072, 0.097) | -0.007 |
| *15-week follow-up* | | | | | | | | |
| Configural Model | 584.321 (68) *** |  | 0.990 |  | 0.986 |  | 0.178 (0.165, 0.192) |  |
| Threshold Model | 602.519 (74) *** | 4.052 (6) | 0.990 | 0.000 | 0.987 | 0.001 | 0.173 (0.160, 0.186) | -0.005 |
| Metric Model | 607.605 (82) *** | 6.721 (8) | 0.990 | 0.000 | 0.989 | 0.001 | 0.164 (0.152, 0.176) | -0.009 |
| Scalar Model | 631.184 (90) *** | 5.999 (8) | 0.989 | 0.000 | 0.989 | 0.001 | 0.158 (0.147, 0.170) | -0.005 |
| Strict Model | 648.555 (100) *** | 26.442 (10) ** | 0.989 | 0.000 | 0.990 | 0.001 | 0.151 (0.140, 0.163) | -0.007 |
| Threshold |  |  | ≥ 0.900 | ≤ 0.010 | ≥ 0.900 | ≤ 0.010 | ≤ 0.080 | ≤ 0.015 |

The bold type represents the classification. The italics represent the measure time

*Abbreviations:* *χ^2^* Chi-square, *df* degrees of freedom, *CFI* comparative fit index, *TLI* Tucker-Lewis index, *RMSEA* root mean square error of approximation, *Δ* a change in *χ^2^*, *df,* CFI, TLI, and RMSEA

****P* < 0.001

***P* < 0.010

**P* < 0.050

**Table S4** The average variance extracted and construct reliability of the RSES-7 in convergent validity

|  | Baseline | |  | 1-week follow-up | |  | 15-week follow-up | |
| --- | --- | --- | --- | --- | --- | --- | --- | --- |
|  | Positive | Negative |  | Positive | Negative |  | Positive | Negative |
| AVE | 0.640 | 0.799 |  | 0.770 | 0.866 |  | 0.735 | 0.770 |
| CR | 0.825 | 0.808 |  | 0.875 | 0.846 |  | 0.859 | 0.784 |

*Abbreviations: AVE* average variance extracted, *CR* construct reliability

**Table S5** Internal consistency and test–retest reliability: RSES-10 and SRHQ

| Variables | RSES-10 | | |  | SRHQ | | |
| --- | --- | --- | --- | --- | --- | --- | --- |
|  | Global | Positive | Negative |  | Global | Self-Phy | Self-Psy |
| Cronbach’s α (95% CI) | | | | | | | |
| Baseline | 0.911 (0.900, 0.923) | 0.897 (0.882, 0.911) | 0.826 (0.802, 0.851) |  | 0.821 | — | — |
| 1-week follow-up | 0.942 (0.934, 0.950) | 0.940 (0.932, 0.949) | 0.886 (0.870, 0.902) |  | 0.857 | — | — |
| 15-week follow-up | 0.929 (0.920, 0.938) | 0.933 (0.923, 0.942) | 0.850 (0.828, 0.871) |  | 0.802 | — | — |
| McDonald’s ω (95% CI) | | | | | | | |
| Baseline | 0.915 (0.904, 0.927) | 0.897 (0.882, 0.911) | 0.839 (0.817, 0.862) |  | — | — | — |
| 1-week follow-up | 0.944 (0.937, 0.952) | 0.940 (0.931, 0.948) | 0.895 (0.880, 0.910) |  | — | — | — |
| 15-week follow-up | 0.933 (0.924, 0.942) | 0.930 (0.921, 0.940) | 0.865 (0.846, 0.884) |  | — | — | — |
| ICC (95% CI) | | | | | | | |
| ICC (T1, T2) | 0.872 (0.840, 0.897) | 0.837 (0.802, 0.866) | 0.790 (0.752, 0.823) |  | 0.710 (0.658, 0.754) | 0.637 (0.580, 0.688) | 0.693 (0.640, 0.739) |
| ICC (T2, T3) | 0.824 (0.789, 0.853) | 0.793 (0.757, 0.824) | 0.750 (0.704, 0.789) |  | 0.603 (0.543, 0.657) | 0.521 (0.453, 0.583) | 0.565 (0.501, 0.623) |
| ICC (T1, T3) | 0.744 (0.660, 0.803) | 0.743 (0.679, 0.793) | 0.642 (0.564, 0.706) |  | 0.565 (0.500, 0.624) | 0.545 (0.479, 0.605) | 0.512 (0.441, 0.576) |
| SEM | | | | | | | |
| SEM (T1, T2) | 1.607 | 0.921 | 1.209 |  | 0.634 | 0.372 | 0.404 |
| SEM (T2, T3) | 1.915 | 1.045 | 1.329 |  | 0.693 | 0.418 | 0.433 |
| SEM (T1, T3) | 2.237 | 1.131 | 1.558 |  | 0.770 | 0.430 | 0.467 |

This table shows ordinal forms of Cronbach’s α and McDonald’s ω. Standard error of measurement was calculated as “SD × sqrt (1-ICC)”. The McDonald’s ω and the 95% confidential interval of Cronbach’s α cannot be calculated due to the subscales containing only one item

*Abbreviations: RSES* Rosenberg Self-Esteem Scale, *SRHQ* Self-Rated Health Questionnaire, *ICC* intraclass correlation coefficient, *SEM* standard error of measurement, *Self-Phy* Self‑Rated Physical Condition, *Self-Psy* Self‑Rated Psychological Condition
